# The Molecular Biology of Susceptibility to Post-Traumatic Stress Disorder: Highlights of Epigenetics and Epigenomics

**DOI:** 10.3390/ijms221910743

**Published:** 2021-10-04

**Authors:** Ghazi I. Al Jowf, Clara Snijders, Bart P. F. Rutten, Laurence de Nijs, Lars M. T. Eijssen

**Affiliations:** 1 Department of Psychiatry and Neuropsychology, School for Mental Health and Neuroscience (MHeNs), Faculty of Health, Medicine and Life Sciences, Maastricht University Medical Centre, 6200 MD Maastricht, The Netherlands; ghazi.aljowf@maastrichtuniversity.nl (G.I.A.J.); c.snijders@maastrichtuniversity.nl (C.S.); b.rutten@maastrichtuniversity.nl (B.P.F.R.); 2 Department of Public Health, College of Applied Medical Sciences, King Faisal University, Al-Ahsa 31982, Saudi Arabia; 3 European Graduate School of Neuroscience, Maastricht University, 6200 MD Maastricht, The Netherlands; 4 Department of Bioinformatics—BiGCaT, School of Nutrition and Translational Research in Metabolism (NUTRIM), Faculty of Health, Medicine and Life Sciences, Maastricht University, 6200 MD Maastricht, The Netherlands

**Keywords:** epigenetics, DNA methylation, PTSD, traumatic stress, neurobiology, trauma, resilience

## Abstract

Exposure to trauma is one of the most important and prevalent risk factors for mental and physical ill-health. Excessive or prolonged stress exposure increases the risk of a wide variety of mental and physical symptoms. However, people differ strikingly in their susceptibility to develop signs and symptoms of mental illness after traumatic stress. Post-traumatic stress disorder (PTSD) is a debilitating disorder affecting approximately 8% of the world’s population during their lifetime, and typically develops after exposure to a traumatic event. Despite that exposure to potentially traumatizing events occurs in a large proportion of the general population, about 80–90% of trauma-exposed individuals do not develop PTSD, suggesting an inter-individual difference in vulnerability to PTSD. While the biological mechanisms underlying this differential susceptibility are unknown, epigenetic changes have been proposed to underlie the relationship between exposure to traumatic stress and the susceptibility to develop PTSD. Epigenetic mechanisms refer to environmentally sensitive modifications to DNA and RNA molecules that regulate gene transcription without altering the genetic sequence itself. In this review, we provide an overview of various molecular biological, biochemical and physiological alterations in PTSD, focusing on changes at the genomic and epigenomic level. Finally, we will discuss how current knowledge may aid us in early detection and improved management of PTSD patients.

## 1. Introduction

Mental disorders have a high economic burden and are known to impact one’s productivity and quality of life [1]. Among them, stress-related mental disorders are common in various societies and can develop after exposure to environmental stressors. Trauma can be defined as a response to a deeply distressing or disturbing event that overwhelms an individual’s ability to cope, causes feelings of helplessness, and diminishes their sense of self along with their ability to feel the full range of emotions and experiences [2]. Traumatic stress typically relates to events that are shocking and emotionally overwhelming, and that may involve actual or threatened death, serious injury, or a threat to physical integrity.

Exposure to traumatic stress is highly prevalent; it has been reported that approximately 40–90% of the general population are exposed to one or more traumatic stressors during their lifetime, with distinct populations being at ultra-high risk for exposure to multiple traumatic stressors (e.g., military and police personnel) [3,4]. It is one of the most important and prevalent risk factors for various mental disorders [5,6], including post-traumatic stress disorder (PTSD), addiction, schizophrenia, chronic fatigue, and depression. Amidst the aforementioned mental disorders, PTSD unarguably remains among the most well-known, due to its uniqueness in having an etiological factor (i.e., the exposure) incorporated into the diagnostic description and possibly also due to its high prevalence in certain populations, such as in the combat veteran population [7]. On a societal level, the economic costs of trauma-related psychiatric disorders in Europe are enormous. For example, the costs for anxiety disorders have been estimated at an approximate EUR 74 million, including over EUR 8 million for PTSD in 2010 [8]. In addition, the annual productivity loss of PTSD patients is estimated at approximately USD 3 billion [4]. As a result, stress-related disorders pose an enormous burden on affected individuals, families and society as a whole. These figures are expected to rise further when projected to the year 2030, making these mental disorders among the top contributors with regard to the worldwide disease burden [9]. Additionally, there currently are no preventive measures to minimize the impact of traumatic stress on health, and PTSD treatment options are limited.

At the clinical level, PTSD has a range of symptoms and/or behavioural alterations such as intrusions, avoidance/numbing, hyperarousal, sensitization to stressors, and negative alterations in cognitions and mood [10]. While the lifetime prevalence for stress-related psychiatric disorders exceeds 40%, the lifetime prevalence for PTSD is approximately 2–12% and is significantly higher in high-risk populations [4,8]. Moreover, there is great variation in individual susceptibility to develop PTSD symptoms after exposure to a traumatic stressor [11]. Indeed, more than 80% of trauma-exposed individuals will remain symptom-free [11]. Evidence accumulates that differential susceptibility to traumatic stress may be related to context-dependent functional and transcriptional “epigenetic” alterations in distinct neuronal circuitries involving the hippocampus and the amygdala, as well as in brain–body interactions including the hypothalamic–pituitary–adrenal axis (HPA axis) and the immune system, amongst others [5,12].

Clinically, the cornerstone of PTSD treatment strategies is formed by exposure-based therapies, such as prolonged imaginary exposure, Eye Movement Desensitization and Reprocessing (EMDR) or narrative exposure therapy (NET) [13], with fear extinction learning as one of the assumed mechanisms mediating a reduction in symptoms, while also more classical forms of cognitive therapies are being applied [13]. Moreover, selective serotonin reuptake inhibitors (SSRIs) are administered, with sertraline and paroxetine as FDA-approved agents, and several other pharmaceuticals applied off-label, including atypical antipsychotic agents [14]. Neuromodulation strategies, including electroconvulsive therapy, transcranial stimulation, cranial nerve stimulation and deep brain stimulation, have been applied experimentally and await additional studies before clinical recommendations can be made for their potential inclusion as additional modes of treatment for patients with (distinct forms of) PTSD [15].

As with many other diseases, both genetic and environmental factors affect the onset, severity, and manifestations of PTSD. By studying epigenetic factors, one can potentially obtain a measure of the impact of environmental insults on genetic expression. Epigenetics refers to DNA modifications caused by environmental changes that regulate gene transcription without altering the genetic sequence itself. Because of the brain’s central role in a person’s dynamic adaptations to environmental exposures, epigenetic research is pertinent in neurosciences and mental health [16,17,18]. Hence, gaining a better understanding of epigenetic modifications caused by traumatic stress would be of great benefit for patients, clinicians and society as a whole. DNA methylation, or the methylation of cytosine residues to 5-methylcytosine (5-mC), is one of the best-characterized epigenetic mechanisms in the mammalian genome and is involved in long-term persistent alterations along with more volatile changes induced by environmental exposures [19,20]. DNA methylation not only programs the identity of cells, but also contributes to the adaptive capacity of the transcriptional response to dynamic alterations in environmental factors throughout life. Other epigenetic mechanisms are also being increasingly studied, including histone modifications and miRNAs. For all of these, in recent years, approaches have moved from targeted gene or genomic region-based ones to genome-wide explorations looking for integrated regulatory patterns or mechanisms in the field of epigenomics.

**Figure 1:**
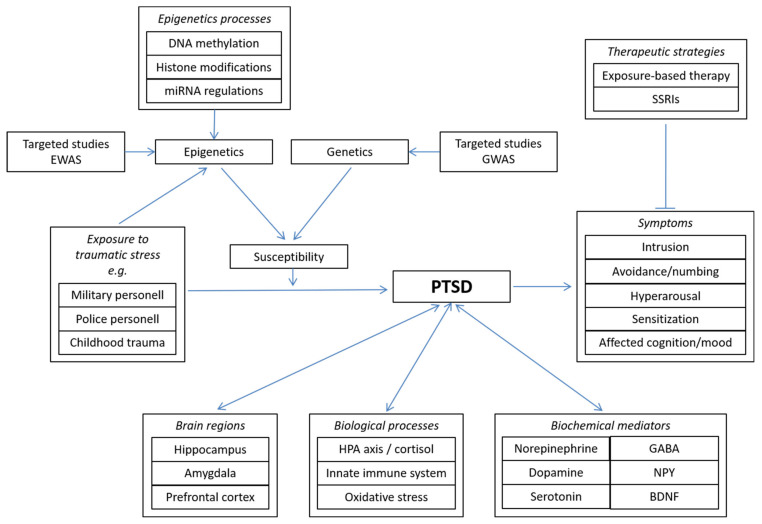
Concept map giving an overview of the information about PTSD discussed in this review.

Therefore, the aim of this narrative review paper is to provide an overview of the current knowledge with respect to certain important aspects and highlights in the fields of neurobiology, epigenetics and epigenomics in relation to PTSD. Figure 1 provides an general overview of the information about PTSD discussed in this review.

## 2. Neurobiology of PTSD

PTSD is a complex pathological entity that evolves over time. It is usually triggered by abnormally high levels of physiological stress that impact neuronal biochemical configurations and neuroplasticity within the central nervous system [21]. In the following sections, we will elaborate on the neurobiological characteristics of PTSD.

### 2.1. Neuroanatomical Changes Associated with PTSD

In recent decades, imaging techniques have been used to visualize the brains of people exposed to trauma. Several studies have reported structural changes in two specific brain areas, i.e., the hippocampus and the anterior cingulate cortex (ACC) [22]. While the hippocampus is involved in memory and learning, the ACC plays a role in reasoning and decision-making [23]. Moreover, functional magnetic resonance imaging (fMRI) has revealed reduced prefrontal cortex activity when people with PTSD are reminded of the trauma, and increased activity of the amygdala, which processes fear and emotion [24]. This contrasts with increased activity in the prefrontal cortex of people who experience trauma without developing PTSD [25,26,27]. Recent research has furthermore indicated that functional differences might also play a role in the development and persistence of stress-induced mental disorders. Research performed by Ressler and colleagues on highly traumatized individuals showed that resilient individuals have better connections between the ACC and the hippocampus in contrast to those suffering from PTSD [28]. This suggests that susceptibility to stress may relate to disturbed communication between the cortical reasoning circuitry and the emotional circuitry of our limbic system.

### 2.2. Biochemical Mediators in PTSD Patients

#### 2.2.1. Norepinephrine

Norepinephrine is a pivotal transmitter in the peripheral and central nervous system. The locus coeruleus, in which the cell somata produce norepinephrine reside, is also targeted by antianxiety drugs, including benzodiazepines and alcohol [29].

Some phenotypes and symptoms of PTSD, including irritability, hyperarousal, and sleep disturbance, have been specifically related to altered noradrenergic function [30], and it has been proposed that the sympathetic function in PTSD individuals may suffer from an uncoupling between the central and peripheral autonomic fields [31]. The α2-norepinephrine receptors have been documented to show particular alterations in stress conditions, although this is likely not specifically linked to PTSD [32]. It has been documented that deficiency of deficient α2-adrenoreceptors is associated with increased vulnerability to stressors in mice [33,34].

#### 2.2.2. Dopamine

Dopamine is another abundant neurotransmitter in the brain, mainly produced in the midbrain region and transported via axons to other brain regions [35]. Evidence for its role in PTSD can be viewed from three different aspects: a molecular aspect, including genetic, ligand–receptor and metabolic alterations, regional alterations, and specific clinical symptoms. While several studies trying to find associations between specific dopamine receptor genes (DRD2) and PTSD showed associations [36,37], others did not [38]. Metabolic alterations of dopamine have been confirmed by finding increased dopamine concentrations in the plasma or urine of PTSD patients [39,40], and an increase in their dopamine beta-hydroxylase activity [41]. Regionally, the medial prefrontal cortex shows a high release of dopamine from axonal projections after stressful conditions [42,43]. Interestingly, cognitive impairment associated with the stress response has been linked to dopamine release in the prefrontal cortex in conjunction with other neurotransmitters [44]. These studies suggest an important role of the dopamine system in the pathophysiology of PTSD.

#### 2.2.3. Serotonin

Serotonin is a neurotransmitter which has widespread actions on behavioural and physiological functions [35]. Human studies show that decreased serotonergic function is associated with PTSD-related symptoms and behaviour such as impulsivity [45,46,47], aggression [46], depression and suicide [48,49]. Genetic associations between PTSD and specific serotonin transporter molecules have been reported. For example, PTSD is associated with the SS (short–short) allele genotype, which leads to lower expression of the serotonin transporter 5-HTT and as such lower serotonin reuptake from the synapse [50,51,52,53,54]. In contrast, the LL (long–long) genotype of the serotonin transporter in PTSD is associated with enhanced response to SSRIs, while the SS allele is associated with non-responsiveness [54,55,56]. The SS genotype in PTSD is also associated with a poor response to cognitive behavioural treatment [57]. These findings highlight the genetic precursors of susceptibility to PTSD associated with serotonin.

#### 2.2.4. Gamma-Aminobutyric Acid (GABA)

GABA is an inhibitory neurotransmitter which is ubiquitously present throughout the brain. Its receptors have been classified in three main classes (GABA-A, GABA-B, and GABA-C). Benzodiazepines are the most important agonists binding to GABA-A receptors. GABA-A receptors show altered (decreased) binding with ligands in the frontal cortex following specific types of stressors [58]. GABA benzodiazepine receptors also showed decreased expression in the hippocampus after prenatal stress [59] and in a fearful strain of rats [60]. Human studies also showed decreased GABA-A binding capacity in Vietnam combat veterans with PTSD [61]. These findings show that alterations in GABA receptor expression or binding capacity may have effects on stress-related mental disorders, with a possible role in PTSD.

#### 2.2.5. Neuropeptide Y (NPY)

NPY is a neuropeptide expressed in various parts of the brain, including the forebrain, limbic system, and brainstem, which regulate emotional and stress behaviours [62]. Interest in NPY took off in 2000 following a study on healthy US Army soldiers [63]. These soldiers participated in a survival course, which was designed to simulate the conditions experienced by prisoners of war [63]. Their serum NPY levels went up within a few hours after exposure to military interrogations during the survival course. Furthermore, most of the soldiers of the Special Forces who had trained to be resilient had significantly increased NPY levels relative to the non-Special Forces or the typical ones [63]. This supports the idea that NPY enhances stress resilience, which is generally seen in humans. Other preclinical and clinical studies corroborate these data, suggesting that reduced NPY in the central nervous system is associated with PTSD [62]. The most common SNP studied for NPY is rs16147 (−399T > C) polymorphism, which is associated with low concentrations of NPY, resulting in hyperarousal in the brainstem, stress activation alterations in the HPA axis and re-experiencing in the hippocampus [62]. Other studies, in addition, found other factors caused by trauma (TNFa) increase dysregulating the HPA axis [64]. The −1002T > G loci are also associated with low NPY concentration in the CSF and amygdala, associated with increased levels of anxiety, arousal, addictive behaviours, and reduced stress resiliency [62].

#### 2.2.6. Brain-Derived Neurotrophic Factor (BDNF)

BDNF, which regulates neuronal survival, growth differentiation, and synapse formation, is known to be involved in PTSD as well as depression [65]. Some studies have shown that BDNF is associated with PTSD risk and exaggerated startle reaction (a major arousal manifestation of PTSD) in the United States military service members who were deployed during the wars in both Iraq and Afghanistan [65]. Furthermore, subjects with PTSD showed higher levels of BDNF in their peripheral blood plasma than the non-PTSD controls. Increased BDNF levels were observed in both blood plasma and hippocampal tissue in the inescapable tail shock rat model of PTSD [65]. This highlights the importance of BDNF as a potential biomarker and its possible roles in the onset of PTSD [65]. Fundamental research shows that stress decreases the expression of BDNF in the hippocampus [66]. This, along with another finding on the effect of stress on inducing neuronal loss and damage in the CA3 region of the hippocampus [67], and the pivotal role of the hippocampus in handling stressful conditions, may point to the fact that stress initially decreases BDNF and subsequently results in hippocampal damage, leading to PTSD.

#### 2.2.7. Oxidative Stress in PTSD

Many studies have reported that increases in oxidative stress might contribute to the development of psychiatric disorders, including PTSD. Stress is considered to be a risk factor for PTSD development and triggers a sustained growth in nitric oxide synthase (NOS) activity, which might generate extreme amounts of nitric oxide. In general, the oxidation of nitric oxide produces a substance called ‘peroxynitrite’, which is extremely toxic to all nerve cells. Observations of high levels of peroxynitrite and its predecessor nitric oxide have been reported in patients with PTSD [68].

### 2.3. Neuroendocrine Changes: Alterations in the HPA Axis

The HPA axis is a hierarchical system comprising the hypothalamic, pituitary and adrenal glands which orchestrates the homeostatic balance of the organism’s response to environmental stress. The effective arm of this axis is cortisol and its analogues, which are stress hormones. Stress hormones modulate the physiological, immunological, and treatment responses in PTSD [69]. There are gender differences in response to traumatic events. Women show a higher incidence of trauma-related psychiatric disorders, including PTSD, than their male counterparts [69]. Animal studies also show a strain-specific difference in fear behaviour in mice, related to the difference in their glucocorticoid response to stressful events [70]. In parallel, human foetuses experiencing severe pregnancy stress will have an altered HPA axis and will have a higher risk of neuropsychiatric disorders later in life [71].

Several studies have aimed to demonstrate a relationship between increases in stress hormone levels and the intensity of an encountered traumatic event. A captivity survival program in the Canadian Armed Forces showed that salivary cortisol and dehydroepiandrosterone (DHEA) increased the most in scenarios with higher-intensity interrogations [72]. Data from adolescents exposed to the 1988 earthquake in Armenia showed a significant relation between adrenocorticotropic hormone (ACTH) response to exercise challenges, and severity of PTSD [73].

Other studies showed a relationship between initial hormonal levels immediately after a stressful event and the risk of subsequent development of PTSD. Higher initial urinary epinephrine and cortisol levels immediately following a traumatic event are associated with an increased risk of developing PTSD later in life in child trauma victims [74]. In contrast, urinary cortisol levels immediately following admission to hospital in motor vehicle accident victims were lower in those who subsequently developed PTSD [75].

Therapeutic approaches can modulate the HPA axis in PTSD patients. A study on PTSD patients showed an increase in serum cortisol and DHEA levels after brief eclectic psychotherapy in those who responded to this treatment modality [76].

## 3. Heritability and Genetic Findings of PTSD

Though it is predicted that up to 90% of individuals may suffer from a significant traumatic experience in their lifetime, PTSD is only found to develop in 20–30% of exposed individuals [77]. Benerjee et al. (2017) indicate that a major contributing factor to this difference could be genetic heritability along with early childhood trauma. This is reflected by twin studies showing 30–40% genetic heritability estimates for PTSD [78]. Genome-wide association studies (GWAS) have addressed some of these genetic factors and identified associations with single-nucleotide polymorphisms (SNP) in certain genes, including the retinoid-related orphan receptor gene *(RORA)*, Neuroligin gene *(NLGN1)* and Tolloid-like 1 gene *(TLL-1)* [19]. The *RORA* gene protein has the potential to modulate neurons to respond to steroid hormone changes, oxidative stress, and inflammation. This modulatory effect is severed by trauma-induced changes of RORA. Furthermore, common variants within the *FKBP5* gene have been shown to increase the risk of developing PTSD and MDD [79,80]. A recent work further studied the genetic underpinning of PTSD in relation to MDD, by applying a multi-omics integrative approach using genomics and transcriptomics data. They identified 13 potential driver genes for PTSD symptoms, with *ESR1, RUNX1, PPARA*, and *WWOX* also driving MDD symptoms and connected to biological pathways which have been linked previously to the regulation of stress and trauma response [81].

Additionally, the *NLGN1* gene is also found to be linked with PTSD and other psychiatric disorders [82]. This gene encodes for synaptic adhesion molecules and thereby has a role in synaptogenesis and synaptic maintenance. NLGN1 depletion in the amygdala in a mouse model of PTSD shows a depletion of fear-associated memory storage, indicating a prominent role in PTSD [82]. Moreover, the *TLL-1* gene that functions in remodelling the extracellular matrix has recently been found to be a contributing gene for the development of PTSD [19]. It is important to consider, however, that there is a conflict in considering GWAS methods while trying to move away from false positives and heterogeneity across samples [83]. Hence, despite the major contribution of GWAS in this field, GWAS approaches to date have not (yet) produced many replicable findings [83].

In 2019, Nievergelt et al. conducted a meta-analysis of PTSD genome-wide association studies, using a sample size of about 200,000 individuals consisting of more than 30,000 PTSD cases and 170,000 controls. The main aim of their study was to demonstrate the genetic risk for PTSD by estimating heritability based on molecular genetic data, to discover genome-wide significant hits linked to PTSD. They found heritability estimates in the range of 5–20% and identified contributing genes, including *PARK2*, *PODXL*, *SH3RF3*, *ZDHHC14*, *KAZN*, as well as several non-coding RNAs [84].

## 4. Epigenetic Changes

Epigenetic mechanisms regulate gene expression levels without altering the DNA sequence [85,86]. These mechanisms include histone modifications, DNA methylation, and post-transcriptional regulation by non-coding RNAs (RNA-associated silencing) such as microRNAs (miRNAs). Epigenetic changes can be acquired over the lifespan and mediate environmental effects on gene expression. In the brain, epigenetic regulation is vital for basic cellular processes involved in multiple aspects of neuronal function, such as synaptic plasticity, and for complex behaviours, such as those involved in learning and memory [87]. Epigenetic mechanisms are affected by various factors and processes, including development (both in utero and during childhood), environmental chemicals, drugs/pharmaceuticals, aging, and diet.

An increasing number of studies show the relevance of epigenetic changes in response to traumatic stress across the life span. These provide a possible link between the environment and gene expression, since stress could have an influence on gene expression by inducing specific changes in epigenetic regulation [88,89,90]. These changes might be different for some individuals who develop PTSD and those who do not. By finding specific epigenetic patterns, scientists could discover diagnostic and/or prognostic biomarkers, and even enhance therapeutic opportunities. In addition, many animal studies have been instrumental in delivering clues for a causal relationship between the early life environment, epigenomic changes, and subsequent behavioural changes.

Although available evidence is limited, transgenerational epigenetic research performed in animals indicates that the epigenetic effects of trauma may be transmitted to multiple generations. Therefore, traumatic stress could already affect the epigenetic background of said offspring during pregnancy [90,91,92,93,94]. If confirmed, this could be predictive for the possible future development of PTSD, especially in light of at-risk jobs where trauma exposure is more prevalent. Below, we describe the roles of the different epigenetic mechanisms, pharmacological aspects, and relationships with other factors.

### 4.1. Histone Modification

Histone proteins can be altered by the addition of one or more chemical groups to one or more residues in the chain, including acetyl, citrulline, methyl, ubiquitin, a small-ubiquitin-like modifier, phosphorus, and ribose [95]. The effect of histone modification on transcription is quite diverse: histone acetylation opens up chromatin conformation to allow transcription, histone phosphorylation often indicates DNA damage, whereas the transcriptional impact of mono-, di-, or tri-methylation is dependent on the modified histone protein and residue [96].

Animal studies that measured brain histone acetylation after fear conditioning [96,97] showed that histone H4 lysine 5 acetylation (H4K5ac) and histone H3 lysine 9 (H3K9ac) were increased significantly in the lateral, basal, and centrolateral amygdala. H3K9ac and H4K5ac were also shown to be increased in the centromedial amygdala and the prelimbic-prefrontal cortex (PL-PFC), but this only occurred after fear learning. Similarly, after fear learning, differential H4K5ac levels were observed in the prefrontal cortex, with a significant decrease in the infralimbic-prefrontal cortex (IL-PFC) and an increase in the PL-PFC. Furthermore, histone acetylation also showed variations after fear extinction in which rat IL-PFC showed significantly higher levels of H3K9ac after delayed extinction compared to no (or immediate) extinction [96,98].

In humans, histone trimethylation variances have been noted in PTSD subjects at various lysine sites in peripheral blood monocytes [96,99]. It is worth noting that several histone modifications are associated with DNA methylation changes in certain genes, as well as expression changes of miRNAs, especially relating to the expression of pro-inflammatory cytokines [96].

### 4.2. DNA Methylation

DNA methylation, mediated by DNA methyltransferases (DNMT), typically reduces or blocks gene transcription and occurs most frequently at the cytosine of a CpG site (a DNA sequence where cytosine is 5-prime to guanine, connected via a phosphate group) [96,100].

A study taking account of genome-wide gene expression and DNA methylation in 12 PTSD subjects as well as 12 trauma-exposed healthy control subjects discovered 3989 genes that were significantly upregulated in those with PTSD and three downregulated (*p* < 0.05), which was adjusted for multiple comparisons. However, there was no significant difference in DNA methylation [96,101]. The same study observed upregulation in olfactory function and immune system gene expression. Other studies also noted downregulation in immune system genes [96]. Other methylome-wide studies have discovered that DNA methylation variation at certain loci, genes and biological processed showed significant association with PTSD, as shown in the EWAS studies mentioned in Section 5.

### 4.3. MicroRNA

RNA-associated silencing is a process whereby non-coding RNAs (nc-RNAs) negatively impact gene expression. Long nc-RNAs (lncRNA) are transcripts that are larger than 200 nucleotides. Short nc-RNAs (sncRNA) appear in a very large range of molecules, and include miRNA, piwi-interacting RNA (piRNA), and small interfering RNA (siRNA) [96].

Hundreds of different miRNAs can be found in humans, which together regulate the activity of 30–60% of all protein-coding genes [102]. miRNAs are transcribed from DNA into primary miRNAs, which are processed further into precursor miRNAs, and finally mature miRNAs [103]. Mature miRNAs most often bind to the 3′ untranslated region (UTR) of target messenger RNAs (mRNAs), thereby affecting gene regulation by silencing and/or suppressing gene expression [102]. By targeting a large number of mRNAs, miRNAs are involved in a wide variety of critical processes, including development, metabolism, growth and differentiation [104]. As such, aberrant expression of miRNA has been associated with several human diseases, including cancers, autoimmune diseases, and inflammatory diseases, as well as in some psychiatric disorders, such as major depressive disorder (MDD) and schizophrenia [104]. Various studies based on animal models of PTSD or clinical patients demonstrate alterations in circulating miRNAs in PTSD subjects [105].

In a mouse model for PTSD, the prefrontal cortex (PFC) miRNA profiles demonstrated that traumatic stress on its own did not influence long-term miRNA expression, when compared to control subjects [96,106]. However, the use of fluoxetine treatment in traumatized mice significantly reduced some miRNAs—especially mmu-miR-1971—in comparison to untreated traumatized mice. This finding in particular is relevant for future studies on the subject of RNA-associated epigenetic mechanisms in PTSD sufferers with comorbid depressive/anxiety disorders, as these kinds of comorbidities are often treated with selective serotonin reuptake inhibitors (SSRIs)—for instance, fluoxetine [96]. Additionally, serotonin has been shown to alter the levels of an miRNA that modulates the expression levels of the serotonin transporter [107].

### 4.4. Epigenetics Based Pharmacology

In recent years, drugs that have been used clinically, such as the anticonvulsant and mood-stabilizing agent valproic acid (VPA), have been shown to act on different aspects of the epigenetic machinery. Epigenetic mechanisms of VPA include regulation through transcription factors, DNA methylation and direct inhibition of histone deacetylation [108]. VPA was also shown to affect DNA methylation along with quetiapine when used in bipolar disorder [109]. Though conventionally used drugs for neurological or psychiatric disorders are classified based on their receptor binding, many of them also have additional epigenetic mechanisms of action [110]. The extent of the contribution of these epigenetic mechanisms relative to their neurotransmitter receptor binding capacity is unknown [111]. Amitriptyline alters DNA methylation and is used to treat PTSD [112]. Imipramine, a histone deacetylase (HDAC) inhibitor, and many other psychiatric drugs, such as paroxetine, citalopram, haloperidol, and clozapine, have been shown to have epigenetic mechanisms [107,110,111,112]. Here, we describe some recent insights into the role of HDAC inhibitors and DNMT inhibitors in more detail.

#### 4.4.1. HDAC Inhibitors

Histone acetyltransferases (HAT) add an acetyl group to the histone proteins, causing the chromatin to relax and allow transcription to take place, leading to the formation of memory. HDAC removes acetyl groups, causing the chromatin to tighten, and hinders transcription [113]. Although both HDAC and HAT can be targeted using distinct compounds, only HDAC inhibitors have thus far been tested in relation to fear processing [114]. The inhibition of HDAC has demonstrated a role in enhancing a number of putatively protective genes and has been linked with neuroprotection and memory formation [115]. When injected before the fear conditioning session, HDAC inhibitors boosted the long-term fear memory formation, but when injected into the IL-PFC during extinction training in mice, they have been shown to boost new memory formation during extinction [116]. This highlights the potential use of HDAC inhibitors in the treatment of PTSD.

VPA, trichostatin A, and sodium or phenylbutyrate are clinically used HDAC inhibitors [113]. VPA in particular is currently used for its role as an anti-convulsant and mood stabilizer. Long-term treatment with VPA has been linked to enhancement of GABAergic signalling [114]. VPA and Vorinostat, also known as suberanilohydroxamic acid, which inhibits histone deacetylase, have both shown to be promising new avenues in treating PTSD and mood symptoms. Nevertheless, a randomized control trial (RCT) should be the next step in order to fully comprehend their efficacy [117,118].

Histone-modifying complexes can further include histone acetyltransferases and HDACs [119]. The delayed onset of the effect of many antidepressant drugs is an example of the possible indirect epigenetic modification effects of these pharmacotherapies. Some psychiatric drugs have a direct effect on enzymes catalysing epigenetic changes. Among them are VPA [120] and the antidepressant tranylcypromine [121].

#### 4.4.2. DNA Methyltransferase (DNMT) Inhibitors

DNMT catalyse the transfer of a methyl group to the genome, generally hindering transcription when occurring in CpG islands. DNMT inhibitors, such as HDAC inhibitors, have shown promising effects on fear extinction by inducing deficits in learning in relation to fear. However, they work in much the opposite way as HDAC inhibitors. Infusion of DNMT inhibitors to the lateral amygdala in rodents promotes the extinction of memory rather than the formation of new memories [113]. 5-aza-2′-deoxycytidine (5-AZA) is one of the few DNMT inhibitors that have been tested in this regard, and it has demonstrated a preventative effect on fear memory formation by increasing acetylation of histones [122].

### 4.5. The Impact of Lifestyle, Psychotherapy, and Behavioural Therapy on Epigenetic Mechanisms

There is accumulating evidence that epigenetics affect the stress, health, and behaviour of an individual, but more importantly, this also works vice versa [123]. Recent studies indicate that behavioural approaches used to treat PTSD such as cognitive behavioural therapy and/or lifestyle modifications such as long-term meditation can alter neurophysiological traits and transcriptional profiles [123]. The study conducted by Kaliman et al. (2014) depicted the dynamic role of behavioural change on epigenetics and its benefits in some PTSD symptoms [124]. This study included 8 h of meditation and within those hours, experienced meditators indicated lower expression of HDAC genes (*HDAC2*, *3*, and *9*). These histone changes could be beneficial to PTSD patients as the downregulation of *HDAC2* is associated with an improved cortisol recovery after stress. Additionally, the intervention group showed a significant reduction in the pro-inflammatory gene *COX-2*, as this gene is dependent on HDAC expression changes as well [124].

## 5. Epigenomic Technology Modern Approach

In contrast to earlier epigenetic research, which was more aimed at demonstrating the existence of epigenetic alterations in disease, generally focussing on individual genes or genomic regions, novel aims are to apply genome-wide screening methods and obtain an integrated understanding of regulatory changes. Here, we describe some avenues of state-of-the-art research approaches.

### 5.1. Recent Developments in Epigenetics of Stress Disorders Research

#### 5.1.1. Modern Technologies Used in Epigenomics PTSD Research

This section will elaborate on the techniques most commonly used to study epigenetic underpinnings of stress-related disorders such as PTSD. Of note, it does not aim to provide a comprehensive overview of the available techniques used to study epigenetic mechanisms. DNA methylation analysis has been among the most popular methods to investigate epigenetic variations [125]. The most conventional method includes the sequencing of PCR-amplified and bisulphite-modified DNA [126].

The most comprehensive method to analyse bisulphite-converted DNA is whole-genome bisulphite sequencing (WGBS). Although this approach allows for an unbiased exploration of methylation patterns across the whole genome, its main limitations are a relatively high cost and high complexity of the generated data. Moreover, since full coverage is usually not required, techniques such as reduced representation bisulphite sequencing (RRBS), in which only specific CpG-rich regions are sequenced, are an interesting and less challenging alternative.

Another widely used alternative includes DNA methylation arrays. For example, the latest microarrays from Illumina allow researchers to interrogate over 850,000 CpG sites using the Infinium Methylation EPIC kit, while previous versions were limited to 27 K and, subsequently, 450 K sites. The genome-wide coverage and high-throughput nature of the EPIC BeadChips make them ideal for epigenome-wide association studies (EWAS) [127].

#### 5.1.2. Epigenome-Wide Association Studies Open New Perspectives

EWAS allow for a hypothesis-free investigation of DNA methylation patterns across the genome that are associated with a particular phenotype. To date, several main studies have identified methylation profiles associated with PTSD (Table 1).

Longitudinal studies highlight associations between the development of PTSD symptoms and the emergence of alterations in DNA methylation profiles [128,129]. Studies using longitudinal designs can reveal specific genomic regions in which DNA methylation patterns dynamically change across a period of stress exposure, and potentially mark susceptibility for PTSD [128].

Using such a design on a cohort of Dutch military members (the Prospective Research In Stress-related Military Operations; PRISMO, N = 93), a study conducted by Rutten et al. (2018) assessed post-deployment methylation profiles associated with PTSD status while correcting for pre-deployment measures [128]. Using Illumina HumanMethylation450 BeadChips, the authors identified 17 loci and 12 genomic regions which underwent methylation changes that were associated with PTSD symptoms [128]. Replication analyses in a cohort of US Marine soldiers (N = 98) showed nominal replication for three genes, i.e., *HIST1H2APS2*, *RNF39,* and *ZFP57*. Decreasing DNA methylation levels at these sites were related to increasing levels of PTSD symptoms [128]. Interestingly, when comparing the epigenetic alterations between the first dataset (N = 93) of the Dutch military and the second dataset (N = 98) of the US Marines, the gene region HIST1H2APS2 was one of the only mutual gene positions that had a decrease in methylation in both group sets and increased symptoms of PTSD [128].

Many recent studies have examined global DNA methylation on a genome-wide scale on different groups who have experienced similar traumas (wars, combat, child abuse, veterans, etc.) [64,128,129,130,131,132]. Based on this, one could assume that due to these major commonalities in the studied populations, some CpG sites should be inconsistent throughout all of the studies. This is especially true if these studies claim that epigenetic changes in certain genes could be a biomarker of PTSD development [130,131,132]. However, the *DOCK2* gene site was one of the only CpG site that has been found to have significant methylation changes across three studies [130,132,133].

Logue et al. (2020) recently examined a cohort of veterans from the Translational Research Center for TBI and Stress Disorders (TRACTS) and aimed to replicate their findings using data collected from several military cohorts [130]. One genome-wide significant association with PTSD was identified at cg19534438, located within the *G0S2* gene. Locus methylation was shown to be associated with PTSD, as supported by previous evidence. This finding was replicated in their replication cohort. Other genes that showed association include *AHRR* gene, in which decreased methylation was observed upon smoking [130].

Interestingly, genes related to immune response show alterations in PTSD patients [64,129,130,131,132]. Additionally, several studies examining traumatic stress exposure in patients found high associations between epigenetic changes and immune system dysregulation and/or PTSD development [64,131,132]. According to Mehta et al. (2017), who assessed 211 veterans, methylation changes of the *DOCK2* gene that connect to immune dysregulation and development of PTSD symptoms were found [132]. Furthermore, the hypomethylation of several CpG sites located within *TPR* and *AHHR* related to traumatic stress and PTSD were directly linked with immune system complications such as increased inflammation [64,131]. Interestingly, when assessing the cytokine levels in relation to these epigenetic changes, results indicated that the cytokine changes (IL4, IL2, and TNFa) differ according to the subject’s condition (PTSD, child abuse, or total life stress) [64].

This does not necessarily define a causality but signifies the potential for the influence of immune function on PTSD symptoms or alteration of general immune function in reaction to stressful exposures. Among other genes with correlation to PTSD symptoms is *PARK2,* which is involved in dopamine regulation and is associated with Parkinson’s disease [84]. Further studies on DNA methylation patterns in the brain tissue of PTSD patients, in parallel with their blood samples, show that peripheral epigenetic alterations can mirror epigenomic alterations in the brain [130]. This highlights the possibility of finding PTSD biomarkers based on peripheral tissues [130].

Surprisingly, the study by Mehta and colleagues conducted in 2019 was one of the few studies that investigated transgenerational inheritance of epigenetics, studying 299 veterans diagnosed with PTSD. They tested the *FKBP5* gene that has been previously identified in the literature as a transgenerational transmitted gene (parent to child). Their results in sperm showed four significant CpG sites within this gene that were correlated with PTSD [133].

More recently, Snijders and Maihofer et al. (2020) conducted one of the largest EWAS using longitudinal DNA methylation data of three male military cohorts, i.e., Marine Resiliency Study (MRS), Army STARRS and PRISMO (N = 266, 123 with PTSD) [129]. Their combined results point to three differentially methylated positions (DMP) and twelve differentially methylated regions (DMR), four of which were located within the human leukocyte antigen (HLA) region. Genes located within this region are known to have immune-related functions, and dysregulations within the immune system are not uncommon in PTSD [129]. Interestingly, one DMP and one DMR were located within *MAD1L1*, a gene previously associated with PTSD [134]. Along with the study conducted by Rutten et al. (2018), this study encourages the use of longitudinally collected DNA methylation data. Interestingly, it has been found that these epigenetic changes can be reversible once established. Treatment of PTSD seemingly reverses some of the DNA changes that are related to the development of PTSD, as demonstrated by Vinkers and colleagues. They found that successful treatment resulted in methylation changes in 12 different DMRs, of which ZFP57-increased methylation was the most consistent for PTSD symptom improvement [135].

**Table 1:** Main epigenome-wide association studies in PTSD.

Study	Sample	Findings
Rutten et al. (2018) [128]	Dutch military members (N = 93) of the PRISMO cohort	Identified 17 loci and 12 genomic regions that underwent DNA methylation changes associated with PTSD
Rutten et al. (2018) [128]	US marine soldiers (N = 98) of the MRS cohort	Decreasing DNA methylation levels of the regions HIST1H2APS2, RNF39 and ZFP57 were associated with increasing levels of PTSD symptoms, with the first being the only mutual gene position between the two groups
Logue et al. (2020) [130]	Military veterans of the TRACTS cohort (N = 378)	Locus cg19534438 located within the G0S2 gene methylation was shown to be significantly associated with PTSD
Mehta et al. (2017) [132]	War veterans and males from Grady Trauma Project (N = 211)	Methylation changes of the DOCK2 gene that connect to immune dysregulation and development of PTSD symptoms
Mehta et al. (2019) [133]	Australian or New Zealand armed services in Vietnam (N = 299)	Four significant CpG sites (two CpGs within GR and two CpGs within FKBP5) within FKBP5 gene that were correlated with PTSD
Snijders and Maihofer et al. (2020) [129]	MRS, Army STARRS and PRISMO cohorts (N = 266)	Several differentially methylated positions (DMP) were found, four of which were within the (HLA) region, indicating the importance of immune dysregulation in PTSD

### 5.2. Transgenerational Passing of Epigenetic Traits

Epigenetic inheritance points to the transmission of epigenetic markers from parents to their offspring, hence from one generation to the next. The traits of the offspring are affected without alteration of the primary nucleotide sequence of their DNA. Experiments performed in the past showed that there is a reset of the patterns of DNA methylation in the whole mammalian genome in each generation. This suggests that epigenetic information would not necessarily be inherited [136,137]. Nonetheless, the mammalian epigenetic information is usually not erased between generations and epigenetic modifications can be transmitted through the germline after its exposure [136,138,139,140,141].

### 5.3. Identifying miRNAs as Biomarkers of PTSD

The diagnosis of PTSD is based on reported symptoms and psychological history. However, there is no real diagnostic tool that can confirm the diagnosis. There are significant benefits accompanying the identification of a biomarker for the diagnosis of PTSD. Below, we will elaborate on the possible role of miRNAs as a potential biomarker.

Studies have shown that miRNA profiles could serve as ideal disease biomarker candidates due to their relative stability and presence in several peripheral biofluids [142]. The most common assays that are being used to investigate miRNA expression are microarrays, high-throughput sequencing approaches, or quantitative PCR arrays [143,144]. To date, eight studies have aimed to identify blood-based circulating miRNAs associated with PTSD in humans using one of these approaches [4,99,145,146,147,148,149,150]. Of these, one study performed a total RNA discovery study and found just one upregulated miRNA in PTSD cases (miR-21) [147]. Another study performed a targeted search for the implication of miR-15a following childhood trauma [145], while Wingo et al. (2015) found two downregulated miRNAs in PTSD with comorbid depression as compared to healthy controls (miR-212-3p and miR-3130-5p) [150]. The five remaining studies identified several miRNAs which were either up- or downregulated in military personnel [4,99,146,148,149]. However, comparing findings between these studies reveals very little overlap. This may, at least partly, be due to most studies using relatively small sample sizes and different study assays [151]. This further calls for a need for larger studies to replicate some of the current findings.

While circulating miRNAs are protected from degradation by being bound to Argonaute2 proteins or high-density lipoproteins [152,153], a portion is also found packed within vesicles such as exosomes [154]. The finding that exosomes are involved in intercellular communication led to an increase in the number of studies researching the (miRNA) content of blood-based exosomes. Interestingly, exosomes have also been found to cross the blood–brain barrier and carry cell type-specific surface markers [155,156,157]. By specifically isolating peripheral exosomes exhibiting L1CAM (CD171), a transmembrane protein which is strongly enriched in brain tissue, researchers are now able to indirectly gain insights into (patho) physiological mechanisms occurring within the central nervous system [158]. Although, to the best of our knowledge, this method has not yet been applied to psychiatric disorders, the first studies examining the content of blood-based neuron-derived exosomes of patients with Alzheimer’s disease [159,160] show great promise in using these vesicles as biomarkers for cognitive impairment. This in turn opens up new avenues to explore the potential of delivering miRNA mimics or antagomirs to the brain through exosomes.

## 6. Future Perspectives

With advances in technologies, reductions in cost, and developments and standardisation of analytical workflows, we foresee that genome-wide epigenetic screenings will be increasingly applied in PTSD research. The increased resolution of microarray-based screening (EPIC array) and the establishment of sequencing, WGBS, and RRBS in the field, will generate more precise maps of changes in DNA methylation that relate to the development or progression of PTSD. Some specific projected advances include the following: first of all, aside from DNA methylation, the study of other epigenetic marks will increase and substantially improve our knowledge on their implications in PTSD.

In contrast to methylation, 5-hmC markers in promoter regions have been associated with gene activity rather than repression [161]. The brain is the main organ where the probably deliberate and stable presence of DNA hydroxymethylation markers has been established [162]. With respect to measurement technologies, bisulphite sequencing cannot distinguish between cytosine methylation and hydroxymethylation. Modifications of the protocol, as used in oxidative bisulphite sequencing (oxBS) and Tet-assisted bisulphite sequencing (TAB-Seq), allow us to detect both markers separately [163,164]. Specific investigation of DNA hydroxymethylation has already been applied to increase our understanding of other brain diseases, such as Alzheimer’s disease [165]. Doing the same for PTSD will further increase our understanding of this disorder.

Furthermore, another layer can be added to our understanding of PTSD by the study of histone modifications. These marks can be determined in a genome-wide fashion using a technology called ChIP-seq, which stands for chromatin immunoprecipitation sequencing. In short, histones are fixed to the DNA that is fragmented, after which antibodies recognising a specific histone modification are used. Then, secondary antibodies with attached beads are used to separate the DNA fragments bound to the modified histones from the other DNA fragments. Sequencing of the bound fragments then tells us where the histone modifications were present. For PTSD, it has been applied to characterize epigenetic changes in white blood cells for H3 trimethylation at K4, K9, K27, and K36 [99]. Applying it to additional histone modifications, such as acetylation, and in larger cohorts, or even to generated neurons or organoids from PTSD patients as well could increase our understanding of the underlying regulatory mechanisms. Additionally, it could be used to study more individual epigenetic signatures, as has been performed to characterize H3–K4 trimethylation (H3K4me3) changes in cortical neurons in schizophrenia [166].

Another angle that warrants further study is the passing of traumatic stress-induced biological changes across generations. This has been studied in the offspring of war victims, for example by examining the offspring of holocaust survivors [167]. Differences were observed, but unravelling the underlying mechanisms warrants further investigations [167]. Stress-induced epigenetic changes have also been shown to be inherited by offspring and drive such inheritance, as shown for FKBP5 methylation [133,168,169]. However, this is mostly explained by direct exposure in utero or via exposed germ cells, called intergenerational epigenetic inheritance. As such, true epigenetic inheritance without exposure, called transgenerational, can only be studied in third-generation offspring in the female line and second-generation offspring in the male line. For now, experimental studies have not found any evidence for true epigenetic inheritance. A possible path towards studying this with respect to human PTSD would be to examine the grandchildren of holocaust survivors or victims of other wars. However, such studies need to be carefully designed to distinguish epigenetic inheritance from secondary traumatization. Studies would need to collect rich phenotypic information to elucidate the mechanisms and prove or disprove true transgenerational epigenetic inheritance.

Among the many possible contributing factors to the development of PTSD, only a few of them had gained practical utility in the day-to-day care of the affected patients. Most conventional drugs currently used to treat PTSD have common actions among many other stress disorders, do not have enough disease specificity, and are symptom-based, such as the use of antidepressants. Importantly, none of these medications specifically target the underlying genetic or epigenetic pathologies. Proper management of patients merits due consideration of the epigenetic factors and novel research in drug development targeting these complex molecular changes. Increasing mechanistic understanding may thereby help identify subtypes of the disease. Additionally, novel biomarkers may pave the way for more and more targeted preventive measures. Additionally, current preventive measures do not consider possible transgenerational passing of psychiatric traits to the next generations.

In the longer future, elucidating the role of biological alterations, including epigenetic processes in susceptibility to traumatic stress exposure could promote breakthroughs in the development of new drug targets and biomarkers for psychiatric disorders. The identification of predictive biomarkers that may distinguish between individuals at high or low risk of developing PTSD following trauma exposure could enable more effective preventive strategies and early interventions.

## 7. Conclusions

Fundamental research has progressively given insight into the molecular mechanisms and cellular processes underlying the pathophysiology of PTSD.

Among many physiological alterations in PTSD, including the potential presence of blood biomarkers, PTSD is characterized by structural and functional brain changes. In addition, some of the underlying metabolic effects of conventional pharmacotherapies for PTSD have been linked to epigenetic changes, which also occur throughout disease progression.

This points to the fact that future preventive, diagnostic, and therapeutic paradigms must take complex molecular and epigenetic alterations into consideration. This also promotes understanding of this disorder through a more organic rather than a purely psychological perspective.

## Data Availability

Not applicable.

## References

[1] Wittchen H.U., Jacobi F., Rehm J., Gustavsson A., Svensson M., Jönsson B., Olesen J., Allgulander C., Alonso J., Faravelli C. (2011). The size and burden of mental disorders and other disorders of the brain in Europe 2010. Eur. Neuropsychopharmacol..

[2] Giller E. (1999). What Is Psychological Trauma.

[3] De Vries G.J., Olff M. (2009). The lifetime prevalence of traumatic events and posttraumatic stress disorder in the Netherlands. J. Trauma Stress.

[4] Zhou J., Nagarkatti P., Zhong Y., Ginsberg J.P., Singh N.P., Zhang J., Nagarkatti M. (2014). Dysregulation in microRNA expression is associated with alterations in immune functions in combat veterans with post-traumatic stress disorder. PLoS ONE.

[5] Yehuda R., Hoge C.W., McFarlane A.C., Vermetten E., Lanius R.A., Nievergelt C.M., Hobfoll S.E., Koenen K.C., Neylan T.C., Hyman S.E. (2015). Post-traumatic stress disorder. Nat. Rev. Dis. Primers.

[6] Klengel T., Binder E.B. (2015). Epigenetics of Stress-Related Psychiatric Disorders and Gene × Environment Interactions. Neuron.

[7] Steenkamp M.M., Litz B.T., Hoge C.W., Marmar C.R. (2015). Psychotherapy for Military-Related PTSD: A Review of Randomized Clinical Trials. JAMA.

[8] Olesen J., Gustavsson A., Svensson M., Wittchen H.U., Jönsson B. (2012). The economic cost of brain disorders in Europe. Eur. J. Neurol..

[9] Hock R.S., Or F., Kolappa K., Burkey M.D., Surkan P.J., Eaton W.W. (2012). A new resolution for global mental health. Lancet.

[10] Bryant R.A. (2019). Post-traumatic stress disorder: A state-of-the-art review of evidence and challenges. World Psychiatry.

[11] Horn S.R., Charney D.S., Feder A. (2016). Understanding resilience: New approaches for preventing and treating PTSD. Exp. Neurol..

[12] Galatzer-Levy I.R., Steenkamp M.M., Brown A.D., Qian M., Inslicht S., Henn-Haase C., Otte C., Yehuda R., Neylan T.C., Marmar C.R. (2014). Cortisol response to an experimental stress paradigm prospectively predicts long-term distress and resilience trajectories in response to active police service. J. Psychiatr. Res..

[13] Guideline Development Panel for the Treatment of PTSD in Adults, American Psychological Association (2019). Summary of the clinical practice guideline for the treatment of posttraumatic stress disorder (PTSD) in adults. Am. Psychol..

[14] Alexander W. (2012). Pharmacotherapy for post-traumatic stress disorder in combat veterans: Focus on antidepressants and atypical antipsychotic agents. Pharm. Ther..

[15] Koek R.J., Roach J., Athanasiou N., van–t Wout-Frank M., Philip N.S. (2019). Neuromodulatory treatments for post-traumatic stress disorder (PTSD). Prog. Neuro-Psychopharmacol. Biol. Psychiatry.

[16] Nestler E.J. (2012). Epigenetics: Stress makes its molecular mark. Nature.

[17] Dias C., Feng J., Sun H., Shao N.Y., Mazei-Robison M.S., Damez-Werno D., Scobie K., Bagot R., LaBonté B., Ribeiro E. (2014). β-catenin mediates stress resilience through Dicer1/microRNA regulation. Nature.

[18] Peña C.J., Bagot R.C., Labonté B., Nestler E.J. (2014). Epigenetic signaling in psychiatric disorders. J. Mol. Biol..

[19] Heijmans B.T., Tobi E.W., Stein A.D., Putter H., Blauw G.J., Susser E.S., Slagboom P.E., Lumey L.H. (2008). Persistent epigenetic differences associated with prenatal exposure to famine in humans. Proc. Natl. Acad. Sci. USA.

[20] Malan-Müller S., Seedat S., Hemmings S.M. (2014). Understanding posttraumatic stress disorder: Insights from the methylome. Genes Brain Behav..

[21] Sherin J.E., Nemeroff C.B. (2011). Post-traumatic stress disorder: The neurobiological impact of psychological trauma. Dialogues Clin. Neurosci..

[22] Cardenas V.A., Samuelson K., Lenoci M., Studholme C., Neylan T.C., Marmar C.R., Schuff N., Weiner M.W. (2011). Changes in brain anatomy during the course of posttraumatic stress disorder. Psychiatry Res..

[23] Lavin C., Melis C., Mikulan E., Gelormini C., Huepe D., Ibañez A. (2013). The anterior cingulate cortex: An integrative hub for human socially-driven interactions. Front. Neurosci..

[24] Bremner J.D. (2007). Neuroimaging in posttraumatic stress disorder and other stress-related disorders. Neuroimaging Clin. N. Am..

[25] Van Wingen G.A., Geuze E., Caan M.W., Kozicz T., Olabarriaga S.D., Denys D., Vermetten E., Fernández G. (2012). Persistent and reversible consequences of combat stress on the mesofrontal circuit and cognition. Proc. Natl. Acad. Sci. USA.

[26] Kennis M., van Rooij S.J., van den Heuvel M.P., Kahn R.S., Geuze E. (2016). Functional network topology associated with posttraumatic stress disorder in veterans. Neuroimage Clin..

[27] Van Rooij S.J., Kennis M., Vink M., Geuze E. (2016). Predicting Treatment Outcome in PTSD: A Longitudinal Functional MRI Study on Trauma-Unrelated Emotional Processing. Neuropsychopharmacology.

[28] Hughes V. (2012). Stress: The roots of resilience. Nature.

[29] Bremner J.D., Krystal J.H., Southwick S.M., Charney D.S. (1996). Noradrenergic mechanisms in stress and anxiety: I. Preclinical studies. Synapse.

[30] Bremner J.D., Krystal J.H., Southwick S.M., Charney D.S. (1996). Noradrenergic mechanisms in stress and anxiety: II. Clinical studies. Synapse.

[31] Strawn J.R., Ekhator N.N., Horn P.S., Baker D.G., Geracioti T.D. (2004). Blood pressure and cerebrospinal fluid norepinephrine in combat-related posttraumatic stress disorder. Psychosom. Med..

[32] Torda T., Kvetnanský R., Petríková M. (1985). Effect of repeated immobilization stress on central and peripheral adrenoceptors in rats. Endocrinol. Exp..

[33] Lähdesmäki J., Sallinen J., MacDonald E., Kobilka B.K., Fagerholm V., Scheinin M. (2002). Behavioral and neurochemical characterization of alpha(2A)-adrenergic receptor knockout mice. Neuroscience.

[34] Lähdesmäki J., Sallinen J., MacDonald E., Scheinin M. (2004). Alpha2A-adrenoceptors are important modulators of the effects of D-amphetamine on startle reactivity and brain monoamines. Neuropsychopharmacology.

[35] Bremner J.D., Pearce B. (2016). Neurotransmitter, neurohormonal, and Neuropeptidal Function in Stress and PTSD. Posttraumatic Stress Disorder: From Neurobiology to Treatment.

[36] Comings D.E., Muhleman D., Gysin R. (1996). Dopamine D2 receptor (DRD2) gene and susceptibility to posttraumatic stress disorder: A study and replication. Biol. Psychiatry.

[37] Young R.M., Lawford B.R., Noble E.P., Kann B., Wilkie A., Ritchie T., Arnold L., Shadforth S. (2002). Harmful drinking in military veterans with post-traumatic stress disorder: Association with the D2 dopamine receptor A1 allele. Alcohol Alcohol..

[38] Gelernter J., Southwick S., Goodson S., Morgan A., Nagy L., Charney D.S. (1999). No association between D2 dopamine receptor (DRD2) "A" system alleles, or DRD2 haplotypes, and posttraumatic stress disorder. Biol. Psychiatry.

[39] Hamner M.B., Diamond B.I. (1993). Elevated plasma dopamine in posttraumatic stress disorder: A preliminary report. Biol. Psychiatry.

[40] Lemieux A.M., Coe C.L. (1995). Abuse-related posttraumatic stress disorder: Evidence for chronic neuroendocrine activation in women. Psychosom. Med..

[41] Hamner M.B., Gold P.B. (1998). Plasma dopamine beta-hydroxylase activity in psychotic and non-psychotic post-traumatic stress disorder. Psychiatry Res..

[42] Roth R.H., Tam S.Y., Ida Y., Yang J.X., Deutch A.Y. (1988). Stress and the mesocorticolimbic dopamine systems. Ann. N. Y. Acad. Sci..

[43] Deutch A.Y., Roth R.H. (1990). The determinants of stress-induced activation of the prefrontal cortical dopamine system. Prog. Brain Res..

[44] Arnsten A.F. (2000). Stress impairs prefrontal cortical function in rats and monkeys: Role of dopamine D1 and norepinephrine alpha-1 receptor mechanisms. Prog. Brain Res..

[45] De Cuyper H. (1987). (Auto)aggression and serotonin. A review of human data. Acta Psychiatr. Belg..

[46] Brown G.L., Linnoila M.I. (1990). CSF serotonin metabolite (5-HIAA) studies in depression, impulsivity, and violence. J. Clin. Psychiatry.

[47] Wu J., Kramer G.L., Kram M., Steciuk M., Crawford I.L., Petty F. (1999). Serotonin and learned helplessness: A regional study of 5-HT1A, 5-HT2A receptors and the serotonin transport site in rat brain. J. Psychiatr. Res..

[48] Mann J.J., Arango V., Marzuk P.M., Theccanat S., Reis D.J. (1989). Evidence for the 5-HT hypothesis of suicide. A review of post-mortem studies. Br. J. Psychiatry.

[49] Stanley M., Stanley B. (1990). Postmortem evidence for serotonin–s role in suicide. J. Clin. Psychiatry.

[50] Lee H.J., Lee M.S., Kang R.H., Kim H., Kim S.D., Kee B.S., Kim Y.H., Kim Y.K., Kim J.B., Yeon B.K. (2005). Influence of the serotonin transporter promoter gene polymorphism on susceptibility to posttraumatic stress disorder. Depress. Anxiety.

[51] Koenen K.C., Amstadter A.B., Nugent N.R. (2009). Gene-environment interaction in posttraumatic stress disorder: An update. J. Trauma Stress.

[52] Kuzelova H., Ptacek R., Macek M. (2010). The serotonin transporter gene (5-HTT) variant and psychiatric disorders: Review of current literature. Neuro Endocrinol. Lett..

[53] Wang Z., Baker D.G., Harrer J., Hamner M., Price M., Amstadter A. (2011). The relationship between combat-related posttraumatic stress disorder and the 5-HTTLPR/rs25531 polymorphism. Depress. Anxiety.

[54] Kenna G.A., Roder-Hanna N., Leggio L., Zywiak W.H., Clifford J., Edwards S., Kenna J.A., Shoaff J., Swift R.M. (2012). Association of the 5-HTT gene-linked promoter region (5-HTTLPR) polymorphism with psychiatric disorders: Review of psychopathology and pharmacotherapy. Pharmgenomics Pers. Med..

[55] Mushtaq D., Ali A., Margoob M.A., Murtaza I., Andrade C. (2012). Association between serotonin transporter gene promoter-region polymorphism and 4- and 12-week treatment response to sertraline in posttraumatic stress disorder. J. Affect. Disord..

[56] Pettitt A. (2015). Genetic Variations in the Serotonergic System Mediate a Combined, Weakened Response to SSRI Treatment: A Proposed Model. eNeuro.

[57] Bryant R.A., Felmingham K.L., Falconer E.M., Pe Benito L., Dobson-Stone C., Pierce K.D., Schofield P.R. (2010). Preliminary evidence of the short allele of the serotonin transporter gene predicting poor response to cognitive behavior therapy in posttraumatic stress disorder. Biol. Psychiatry.

[58] Weizman R., Weizman A., Kook K.A., Vocci F., Deutsch S.I., Paul S.M. (1989). Repeated swim stress alters brain benzodiazepine receptors measured in vivo. J. Pharmacol. Exp. Ther..

[59] Barros V.G., Rodríguez P., Martijena I.D., Pérez A., Molina V.A., Antonelli M.C. (2006). Prenatal stress and early adoption effects on benzodiazepine receptors and anxiogenic behavior in the adult rat brain. Synapse.

[60] Robertson H.A., Martin I.L., Candy J.M. (1978). Differences in benzodiazepine receptor binding in Maudsley reactive and Maudsley non-reactive rats. Eur. J. Pharmacol..

[61] Bremner J.D., Innis R.B., Southwick S.M., Staib L., Zoghbi S., Charney D.S. (2000). Decreased benzodiazepine receptor binding in prefrontal cortex in combat-related posttraumatic stress disorder. Am. J. Psychiatry.

[62] Sah R., Geracioti T.D. (2013). Neuropeptide Y and posttraumatic stress disorder. Mol. Psychiatry.

[63] Morgan C.A., Wang S., Southwick S.M., Rasmusson A., Hazlett G., Hauger R.L., Charney D.S. (2000). Plasma neuropeptide-Y concentrations in humans exposed to military survival training. Biol. Psychiatry.

[64] Smith A.K., Conneely K.N., Kilaru V., Mercer K.B., Weiss T.E., Bradley B., Tang Y., Gillespie C.F., Cubells J.F., Ressler K.J. (2011). Differential immune system DNA methylation and cytokine regulation in post-traumatic stress disorder. Am. J. Med. Genet. B Neuropsychiatr. Genet..

[65] Zhang L., Li X.X., Hu X.Z. (2016). Post-traumatic stress disorder risk and brain-derived neurotrophic factor Val66Met. World J. Psychiatry.

[66] Smith M.A., Makino S., Kvetnansky R., Post R.M. (1995). Stress and glucocorticoids affect the expression of brain-derived neurotrophic factor and neurotrophin-3 mRNAs in the hippocampus. J. Neurosci..

[67] Stein-Behrens B., Mattson M.P., Chang I., Yeh M., Sapolsky R. (1994). Stress exacerbates neuron loss and cytoskeletal pathology in the hippocampus. J. Neurosci..

[68] Prasad K.N., Bondy S.C. (2015). Common biochemical defects linkage between post-traumatic stress disorders, mild traumatic brain injury (TBI) and penetrating TBI. Brain Res..

[69] Mendoza C., Barreto G.E., Ávila-Rodriguez M., Echeverria V. (2016). Role of neuroinflammation and sex hormones in war-related PTSD. Mol. Cell Endocrinol..

[70] Brinks V., de Kloet E.R., Oitzl M.S. (2008). Strain specific fear behaviour and glucocorticoid response to aversive events: Modelling PTSD in mice. Prog. Brain Res..

[71] Seckl J.R., Meaney M.J. (2006). Glucocorticoid "programming" and PTSD risk. Ann. N. Y. Acad. Sci..

[72] Suurd Ralph C., Vartanian O., Lieberman H.R., Morgan C.A., Cheung B. (2017). The effects of captivity survival training on mood, dissociation, PTSD symptoms, cognitive performance and stress hormones. Int. J. Psychophysiol..

[73] Goenjian A.K., Pynoos R.S., Steinberg A.M., Endres D., Abraham K., Geffner M.E., Fairbanks L.A. (2003). Hypothalamic-pituitary-adrenal activity among Armenian adolescents with PTSD symptoms. J. Trauma Stress.

[74] Delahanty D.L., Nugent N.R., Christopher N.C., Walsh M. (2005). Initial urinary epinephrine and cortisol levels predict acute PTSD symptoms in child trauma victims. Psychoneuroendocrinology.

[75] Delahanty D.L., Raimonde A.J., Spoonster E. (2000). Initial posttraumatic urinary cortisol levels predict subsequent PTSD symptoms in motor vehicle accident victims. Biol. Psychiatry.

[76] Olff M., de Vries G.J., Güzelcan Y., Assies J., Gersons B.P. (2007). Changes in cortisol and DHEA plasma levels after psychotherapy for PTSD. Psychoneuroendocrinology.

[77] Almli L.M., Fani N., Smith A.K., Ressler K.J. (2014). Genetic approaches to understanding post-traumatic stress disorder. Int. J. Neuropsychopharmacol..

[78] Banerjee S.B., Morrison F.G., Ressler K.J. (2017). Genetic approaches for the study of PTSD: Advances and challenges. Neurosci. Lett..

[79] Binder E.B., Bradley R.G., Liu W., Epstein M.P., Deveau T.C., Mercer K.B., Tang Y., Gillespie C.F., Heim C.M., Nemeroff C.B. (2008). Association of FKBP5 polymorphisms and childhood abuse with risk of posttraumatic stress disorder symptoms in adults. JAMA.

[80] Wang Q., Shelton R.C., Dwivedi Y. (2018). Interaction between early-life stress and FKBP5 gene variants in major depressive disorder and post-traumatic stress disorder: A systematic review and meta-analysis. J. Affect. Disord..

[81] Wuchty S., Myers A.J., Ramirez-Restrepo M., Huentelman M., Richolt R., Gould F., Harvey P.D., Michopolous V., Steven J.S., Wingo A.P. (2021). Integration of peripheral transcriptomics, genomics, and interactomics following trauma identifies causal genes for symptoms of post-traumatic stress and major depression. Mol. Psychiatry.

[82] Kilaru V., Iyer S.V., Almli L.M., Stevens J.S., Lori A., Jovanovic T., Ely T.D., Bradley B., Binder E.B., Koen N. (2016). Genome-wide gene-based analysis suggests an association between Neuroligin 1 (NLGN1) and post-traumatic stress disorder. Transl. Psychiatry.

[83] Logue M.W., Amstadter A.B., Baker D.G., Duncan L., Koenen K.C., Liberzon I., Miller M.W., Morey R.A., Nievergelt C.M., Ressler K.J. (2015). The Psychiatric Genomics Consortium Posttraumatic Stress Disorder Workgroup: Posttraumatic Stress Disorder Enters the Age of Large-Scale Genomic Collaboration. Neuropsychopharmacology.

[84] Nievergelt C.M., Maihofer A.X., Klengel T., Atkinson E.G., Chen C.Y., Choi K.W., Coleman J.R.I., Dalvie S., Duncan L.E., Gelernter J. (2019). International meta-analysis of PTSD genome-wide association studies identifies sex- and ancestry-specific genetic risk loci. Nat. Commun..

[85] Waterland R.A. (2006). Epigenetic mechanisms and gastrointestinal development. J. Pediatrics.

[86] Gibney E.R., Nolan C.M. (2010). Epigenetics and gene expression. Heredity.

[87] Day J.J., Sweatt J.D. (2011). Epigenetic modifications in neurons are essential for formation and storage of behavioral memory. Neuropsychopharmacology.

[88] Boks M.P., de Jong N.M., Kas M.J., Vinkers C.H., Fernandes C., Kahn R.S., Mill J., Ophoff R.A. (2012). Current status and future prospects for epigenetic psychopharmacology. Epigenetics.

[89] Mill J., Heijmans B.T. (2013). From promises to practical strategies in epigenetic epidemiology. Nat. Rev. Genet..

[90] Vinkers C.H., Kalafateli A.L., Rutten B.P., Kas M.J., Kaminsky Z., Turner J.D., Boks M.P. (2015). Traumatic stress and human DNA methylation: A critical review. Epigenomics.

[91] Monk C., Spicer J., Champagne F.A. (2012). Linking prenatal maternal adversity to developmental outcomes in infants: The role of epigenetic pathways. Dev. Psychopathol..

[92] Bohacek J., Mansuy I.M. (2015). Molecular insights into transgenerational non-genetic inheritance of acquired behaviours. Nat. Rev. Genet..

[93] Nagy C., Turecki G. (2015). Transgenerational epigenetic inheritance: An open discussion. Epigenomics.

[94] Nestler E.J. (2016). Transgenerational Epigenetic Contributions to Stress Responses: Fact or Fiction?. PLoS Biol..

[95] Bannister A.J., Kouzarides T. (2011). Regulation of chromatin by histone modifications. Cell Res..

[96] Blacker C.J., Frye M.A., Morava E., Kozicz T., Veldic M. (2019). A Review of Epigenetics of PTSD in Comorbid Psychiatric Conditions. Genes.

[97] Siddiqui S.A., Singh S., Ranjan V., Ugale R., Saha S., Prakash A. (2017). Enhanced Histone Acetylation in the Infralimbic Prefrontal Cortex is Associated with Fear Extinction. Cell Mol. Neurobiol..

[98] Singh S., Siddiqui S.A., Tripathy S., Kumar S., Saha S., Ugale R., Modi D.R., Prakash A. (2018). Decreased level of histone acetylation in the infralimbic prefrontal cortex following immediate extinction may result in deficit of extinction memory. Brain Res. Bull..

[99] Bam M., Yang X., Zhou J., Ginsberg J.P., Leyden Q., Nagarkatti P.S., Nagarkatti M. (2016). Evidence for Epigenetic Regulation of Pro-Inflammatory Cytokines, Interleukin-12 and Interferon Gamma, in Peripheral Blood Mononuclear Cells from PTSD Patients. J. Neuroimmune Pharmacol..

[100] Kulis M., Merkel A., Heath S., Queirós A.C., Schuyler R.P., Castellano G., Beekman R., Raineri E., Esteve A., Clot G. (2015). Whole-genome fingerprint of the DNA methylome during human B cell differentiation. Nat. Genet..

[101] Chen Y., Li X., Kobayashi I., Tsao D., Mellman T.A. (2016). Expression and methylation in posttraumatic stress disorder and resilience; evidence of a role for odorant receptors. Psychiatry Res..

[102] Hammond S.M. (2015). An overview of microRNAs. Adv. Drug Deliv. Rev..

[103] O’Brien J., Hayder H., Zayed Y., Peng C. (2018). Overview of MicroRNA Biogenesis, Mechanisms of Actions, and Circulation. Front. Endocrinol..

[104] Ardekani A.M., Naeini M.M. (2010). The Role of MicroRNAs in Human Diseases. Avicenna J. Med. Biotechnol..

[105] Snijders C., de Nijs L., Baker D.G., Hauger R.L., van den Hove D., Kenis G., Nievergelt C.M., Boks M.P., Vermetten E., Gage F.H. (2018). MicroRNAs in Post-traumatic Stress Disorder. Curr. Top. Behav. Neurosci..

[106] Schmidt U., Herrmann L., Hagl K., Novak B., Huber C., Holsboer F., Wotjak C.T., Buell D.R. (2013). Therapeutic Action of Fluoxetine is Associated with a Reduction in Prefrontal Cortical miR-1971 Expression Levels in a Mouse Model of Posttraumatic Stress Disorder. Front. Psychiatry.

[107] Baudry A., Mouillet-Richard S., Schneider B., Launay J.M., Kellermann O. (2010). miR-16 targets the serotonin transporter: A new facet for adaptive responses to antidepressants. Science.

[108] Boyadjieva N., Varadinova M. (2012). Epigenetics of psychoactive drugs. J. Pharm. Pharmacol..

[109] Houtepen L.C., van Bergen A.H., Vinkers C.H., Boks M.P. (2016). DNA methylation signatures of mood stabilizers and antipsychotics in bipolar disorder. Epigenomics.

[110] Menke A., Klengel T., Binder E.B. (2012). Epigenetics, depression and antidepressant treatment. Curr. Pharm. Des..

[111] Hunter R.G. (2014). Epigenetics in Posttraumatic Stress Disorder. Epigenetics in Psychiatry.

[112] Zimmermann N., Zschocke J., Perisic T., Yu S., Holsboer F., Rein T. (2012). Antidepressants inhibit DNA methyltransferase 1 through reducing G9a levels. Biochem. J..

[113] Pizzimenti C.L., Lattal K.M. (2015). Epigenetics and memory: Causes, consequences and treatments for post-traumatic stress disorder and addiction. Genes Brain Behav..

[114] Whittle N., Singewald N. (2014). HDAC inhibitors as cognitive enhancers in fear, anxiety and trauma therapy: Where do we stand?. Biochem. Soc. Trans..

[115] Narayan P., Dragunow M. (2010). Pharmacology of epigenetics in brain disorders. Br. J. Pharmacol..

[116] Marek R., Coelho C.M., Sullivan R.K., Baker-Andresen D., Li X., Ratnu V., Dudley K.J., Meyers D., Mukherjee C., Cole P.A. (2011). Paradoxical enhancement of fear extinction memory and synaptic plasticity by inhibition of the histone acetyltransferase p300. J. Neurosci..

[117] Adamou M., Puchalska S., Plummer W., Hale A.S. (2007). Valproate in the treatment of PTSD: Systematic review and meta analysis. Curr. Med. Res. Opin..

[118] Fujita Y., Yamamoto S., Morinobu S. (2012). [Novel therapeutic approach for the treatment of post-traumatic stress disorder (PTSD): Facilitating fear extinction]. Nihon Shinkei Seishin Yakurigaku Zasshi.

[119] De Ruijter A.J., van Gennip A.H., Caron H.N., Kemp S., van Kuilenburg A.B. (2003). Histone deacetylases (HDACs): Characterization of the classical HDAC family. Biochem. J..

[120] Phiel C.J., Zhang F., Huang E.Y., Guenther M.G., Lazar M.A., Klein P.S. (2001). Histone deacetylase is a direct target of valproic acid, a potent anticonvulsant, mood stabilizer, and teratogen. J. Biol. Chem..

[121] Lee M.G., Wynder C., Schmidt D.M., McCafferty D.G., Shiekhattar R. (2006). Histone H3 lysine 4 demethylation is a target of nonselective antidepressive medications. Chem. Biol..

[122] Monsey M.S., Ota K.T., Akingbade I.F., Hong E.S., Schafe G.E. (2011). Epigenetic alterations are critical for fear memory consolidation and synaptic plasticity in the lateral amygdala. PLoS ONE.

[123] Kaliman P. (2019). Epigenetics and meditation. Curr. Opin. Psychol..

[124] Kaliman P., Alvarez-López M.J., Cosín-Tomás M., Rosenkranz M.A., Lutz A., Davidson R.J. (2014). Rapid changes in histone deacetylases and inflammatory gene expression in expert meditators. Psychoneuroendocrinology.

[125] Murgatroyd C. (2021). Laboratory techniques in psychiatric epigenetics. Epigenetics in Psychiatry.

[126] Frommer M., McDonald L.E., Millar D.S., Collis C.M., Watt F., Grigg G.W., Molloy P.L., Paul C.L. (1992). A genomic sequencing protocol that yields a positive display of 5-methylcytosine residues in individual DNA strands. Proc. Natl. Acad. Sci. USA.

[127] Mehta D., Klengel T., Conneely K.N., Smith A.K., Altmann A., Pace T.W., Rex-Haffner M., Loeschner A., Gonik M., Mercer K.B. (2013). Childhood maltreatment is associated with distinct genomic and epigenetic profiles in posttraumatic stress disorder. Proc. Natl. Acad. Sci. USA.

[128] Rutten B.P.F., Vermetten E., Vinkers C.H., Ursini G., Daskalakis N.P., Pishva E., de Nijs L., Houtepen L.C., Eijssen L., Jaffe A.E. (2018). Longitudinal analyses of the DNA methylome in deployed military servicemen identify susceptibility loci for post-traumatic stress disorder. Mol. Psychiatry.

[129] Snijders C., Maihofer A.X., Ratanatharathorn A., Baker D.G., Boks M.P., Geuze E., Jain S., Kessler R.C., Pishva E., Risbrough V.B. (2020). Longitudinal epigenome-wide association studies of three male military cohorts reveal multiple CpG sites associated with post-traumatic stress disorder. Clin. Epigenetics.

[130] Logue M.W., Miller M.W., Wolf E.J., Huber B.R., Morrison F.G., Zhou Z., Zheng Y., Smith A.K., Daskalakis N.P., Ratanatharathorn A. (2020). An epigenome-wide association study of posttraumatic stress disorder in US veterans implicates several new DNA methylation loci. Clin. Epigenetics.

[131] Smith A.K., Ratanatharathorn A., Maihofer A.X., Naviaux R.K., Aiello A.E., Amstadter A.B., Ashley-Koch A.E., Baker D.G., Beckham J.C., Boks M.P. (2019). Epigenome-wide meta-analysis of PTSD across 10 military and civilian cohorts identifies novel methylation loci. BioRxiv.

[132] Mehta D., Bruenig D., Carrillo-Roa T., Lawford B., Harvey W., Morris C.P., Smith A.K., Binder E.B., Young R.M., Voisey J. (2017). Genomewide DNA methylation analysis in combat veterans reveals a novel locus for PTSD. Acta Psychiatr. Scand..

[133] Mehta D., Pelzer E.S., Bruenig D., Lawford B., McLeay S., Morris C.P., Gibson J.N., Young R.M., Voisey J. (2019). DNA methylation from germline cells in veterans with PTSD. J. Psychiatr. Res..

[134] Gelernter J., Sun N., Polimanti R., Pietrzak R., Levey D.F., Bryois J., Lu Q., Hu Y., Li B., Radhakrishnan K. (2019). Genome-wide association study of post-traumatic stress disorder reexperiencing symptoms in >165,000 US veterans. Nat. Neurosci..

[135] Vinkers C.H., Geuze E., van Rooij S.J., Kennis M., Schür R.R., Nispeling D.M., Smith A.K., Nievergelt C.M., Uddin M., Rutten B.P. (2021). Successful treatment of post-traumatic stress disorder reverses DNA methylation marks. Mol. Psychiatry.

[136] Bond D.M., Finnegan E.J. (2007). Passing the message on: Inheritance of epigenetic traits. Trends Plant Sci..

[137] Monk M. (1995). Epigenetic programming of differential gene expression in development and evolution. Dev. Genet..

[138] Morgan H.D., Sutherland H.G., Martin D.I., Whitelaw E. (1999). Epigenetic inheritance at the agouti locus in the mouse. Nat. Genet..

[139] Anway M.D., Cupp A.S., Uzumcu M., Skinner M.K. (2005). Epigenetic transgenerational actions of endocrine disruptors and male fertility. Science.

[140] Rakyan V.K., Chong S., Champ M.E., Cuthbert P.C., Morgan H.D., Luu K.V., Whitelaw E. (2003). Transgenerational inheritance of epigenetic states at the murine Axin(Fu) allele occurs after maternal and paternal transmission. Proc. Natl. Acad. Sci. USA.

[141] Cropley J.E., Suter C.M., Beckman K.B., Martin D.I. (2006). Germ-line epigenetic modification of the murine A vy allele by nutritional supplementation. Proc. Natl. Acad. Sci. USA.

[142] Wang J., Chen J., Sen S. (2016). MicroRNA as Biomarkers and Diagnostics. J. Cell. Physiol..

[143] Androvic P., Valihrach L., Elling J., Sjoback R., Kubista M. (2017). Two-tailed RT-qPCR: A novel method for highly accurate miRNA quantification. Nucleic Acids Res..

[144] Nair V.S., Pritchard C.C., Tewari M., Ioannidis J.P. (2014). Design and Analysis for Studying microRNAs in Human Disease: A Primer on -Omic Technologies. Am. J. Epidemiol..

[145] Volk N., Pape J.C., Engel M., Zannas A.S., Cattane N., Cattaneo A., Binder E.B., Chen A. (2016). Amygdalar MicroRNA-15a Is Essential for Coping with Chronic Stress. Cell Rep..

[146] Bam M., Yang X., Zumbrun E.E., Zhong Y., Zhou J., Ginsberg J.P., Leyden Q., Zhang J., Nagarkatti P.S., Nagarkatti M. (2016). Dysregulated immune system networks in war veterans with PTSD is an outcome of altered miRNA expression and DNA methylation. Sci. Rep..

[147] Guardado P., Olivera A., Rusch H.L., Roy M., Martin C., Lejbman N., Lee H., Gill J.M. (2016). Altered gene expression of the innate immune, neuroendocrine, and nuclear factor-kappa B (NF-κB) systems is associated with posttraumatic stress disorder in military personnel. J. Anxiety Disord..

[148] Martin C.G., Kim H., Yun S., Livingston W., Fetta J., Mysliwiec V., Baxter T., Gill J.M. (2017). Circulating miRNA associated with posttraumatic stress disorder in a cohort of military combat veterans. Psychiatry Res..

[149] Snijders C., Krauskopf J., Pishva E., Eijssen L., Machiels B., Kleinjans J., Kenis G., van den Hove D., Kim M.O., Boks M.P.M. (2019). Circulating Serum MicroRNAs as Potential Diagnostic Biomarkers of Posttraumatic Stress Disorder: A Pilot Study. Front. Genet..

[150] Wingo A.P., Almli L.M., Stevens J.S., Klengel T., Uddin M., Li Y., Bustamante A.C., Lori A., Koen N., Stein D.J. (2015). DICER1 and microRNA regulation in post-traumatic stress disorder with comorbid depression. Nat. Commun..

[151] Daskalakis N.P., Provost A.C., Hunter R.G., Guffanti G. (2018). Noncoding RNAs: Stress, Glucocorticoids, and Posttraumatic Stress Disorder. Biol. Psychiatry.

[152] Arroyo J.D., Chevillet J.R., Kroh E.M., Ruf I.K., Pritchard C.C., Gibson D.F., Mitchell P.S., Bennett C.F., Pogosova-Agadjanyan E.L., Stirewalt D.L. (2011). Argonaute2 complexes carry a population of circulating microRNAs independent of vesicles in human plasma. Proc. Natl. Acad. Sci. USA.

[153] Vickers K.C., Palmisano B.T., Shoucri B.M., Shamburek R.D., Remaley A.T. (2011). MicroRNAs are transported in plasma and delivered to recipient cells by high-density lipoproteins. Nat. Cell Biol..

[154] Sato-Kuwabara Y., Melo S.A., Soares F.A., Calin G.A. (2015). The fusion of two worlds: Non-coding RNAs and extracellular vesicles--diagnostic and therapeutic implications (Review). Int. J. Oncol..

[155] Samanta S., Rajasingh S., Drosos N., Zhou Z., Dawn B., Rajasingh J. (2018). Exosomes: New molecular targets of diseases. Acta Pharmacol. Sin..

[156] Larssen P., Wik L., Czarnewski P., Eldh M., Löf L., Ronquist K.G., Dubois L., Freyhult E., Gallant C.J., Oelrich J. (2017). Tracing Cellular Origin of Human Exosomes Using Multiplex Proximity Extension Assays. Mol. Cell Proteom..

[157] Castillo J., Bernard V., San Lucas F.A., Allenson K., Capello M., Kim D.U., Gascoyne P., Mulu F.C., Stephens B.M., Huang J. (2018). Surfaceome profiling enables isolation of cancer-specific exosomal cargo in liquid biopsies from pancreatic cancer patients. Ann. Oncol..

[158] Mustapic M., Eitan E., Werner J.K., Berkowitz S.T., Lazaropoulos M.P., Tran J., Goetzl E.J., Kapogiannis D. (2017). Plasma Extracellular Vesicles Enriched for Neuronal Origin: A Potential Window into Brain Pathologic Processes. Front. Neurosci..

[159] Sun B., Dalvi P., Abadjian L., Tang N., Pulliam L. (2017). Blood neuron-derived exosomes as biomarkers of cognitive impairment in HIV. Aids.

[160] Winston C.N., Goetzl E.J., Akers J.C., Carter B.S., Rockenstein E.M., Galasko D., Masliah E., Rissman R.A. (2016). Prediction of conversion from mild cognitive impairment to dementia with neuronally derived blood exosome protein profile. Alzheimer’s Dement. Diagn. Assess. Dis. Monit..

[161] Ficz G., Branco M.R., Seisenberger S., Santos F., Krueger F., Hore T.A., Marques C.J., Andrews S., Reik W. (2011). Dynamic regulation of 5-hydroxymethylcytosine in mouse ES cells and during differentiation. Nature.

[162] Hahn M.A., Qiu R., Wu X., Li A.X., Zhang H., Wang J., Jui J., Jin S.G., Jiang Y., Pfeifer G.P. (2013). Dynamics of 5-hydroxymethylcytosine and chromatin marks in Mammalian neurogenesis. Cell Rep..

[163] Booth M.J., Ost T.W., Beraldi D., Bell N.M., Branco M.R., Reik W., Balasubramanian S. (2013). Oxidative bisulfite sequencing of 5-methylcytosine and 5-hydroxymethylcytosine. Nat. Protoc..

[164] Yu M., Han D., Hon G.C., He C. (2018). Tet-Assisted Bisulfite Sequencing (TAB-seq). Methods Mol. Biol..

[165] Lardenoije R., Roubroeks J.A.Y., Pishva E., Leber M., Wagner H., Iatrou A., Smith A.R., Smith R.G., Eijssen L.M.T., Kleineidam L. (2019). Alzheimer’s disease-associated (hydroxy)methylomic changes in the brain and blood. Clin. Epigenetics.

[166] Gusev F.E., Reshetov D.A., Mitchell A.C., Andreeva T.V., Dincer A., Grigorenko A.P., Fedonin G., Halene T., Aliseychik M., Filippova E. (2019). Chromatin profiling of cortical neurons identifies individual epigenetic signatures in schizophrenia. Transl. Psychiatry.

[167] Shrira A., Ayalon L., Bensimon M., Bodner E., Rosenbloom T., Yadid G. (2017). Parental Post-traumatic Stress Disorder Symptoms Are Related to Successful Aging in Offspring of Holocaust Survivors. Front. Psychol..

[168] Yehuda R., Daskalakis N.P., Bierer L.M., Bader H.N., Klengel T., Holsboer F., Binder E.B. (2016). Holocaust Exposure Induced Intergenerational Effects on FKBP5 Methylation. Biol. Psychiatry.

[169] Youssef N.A., Lockwood L., Su S., Hao G., Rutten B.P.F. (2018). The Effects of Trauma, with or without PTSD, on the Transgenerational DNA Methylation Alterations in Human Offsprings. Brain Sci..

